# Spatial model of Ebola outbreaks contained by behavior change

**DOI:** 10.1371/journal.pone.0264425

**Published:** 2022-03-14

**Authors:** Gustav S. Halvorsen, Lone Simonsen, Kim Sneppen

**Affiliations:** 1 Niels Bohr Institute, Copenhagen, Denmark; 2 Department of Science and Environment, Roskilde University, Roskilde, Denmark; Universidad Rey Juan Carlos, SPAIN

## Abstract

The West African Ebola (2014-2016) epidemic caused an estimated 11.310 deaths and massive social and economic disruption. The epidemic was comprised of many local outbreaks of varying sizes. However, often local outbreaks recede before the arrival of international aid or susceptible depletion. We modeled Ebola virus transmission under the effect of behavior changes acting as a local inhibitor. A spatial model is used to simulate Ebola epidemics. Our findings suggest that behavior changes can explain why local Ebola outbreaks recede before substantial international aid was mobilized during the 2014-2016 epidemic.

## 1 Introduction

The West African Ebola epidemic was the deadliest outbreak of Ebola virus disease reported to this date. On March 23, 2014, local authorities notified the World Health Organization of an Ebola virus outbreak in southern Guinea. The virus quickly spread to neighboring countries, Liberia and Sierra Leone, causing a net count of 28.616 infections and 11.310 fatalities before ending in June 2016 [1]. The unprecedented scale of the epidemic resulted from dysfunctional healthcare systems, low trust in government following years of armed conflict, and a slow response to the crisis [2]. Risky cultural practices also compounded the severity of the outbreak, particularly burial rites that involve close contact with deceased Ebola patients.

**Fig 1:**
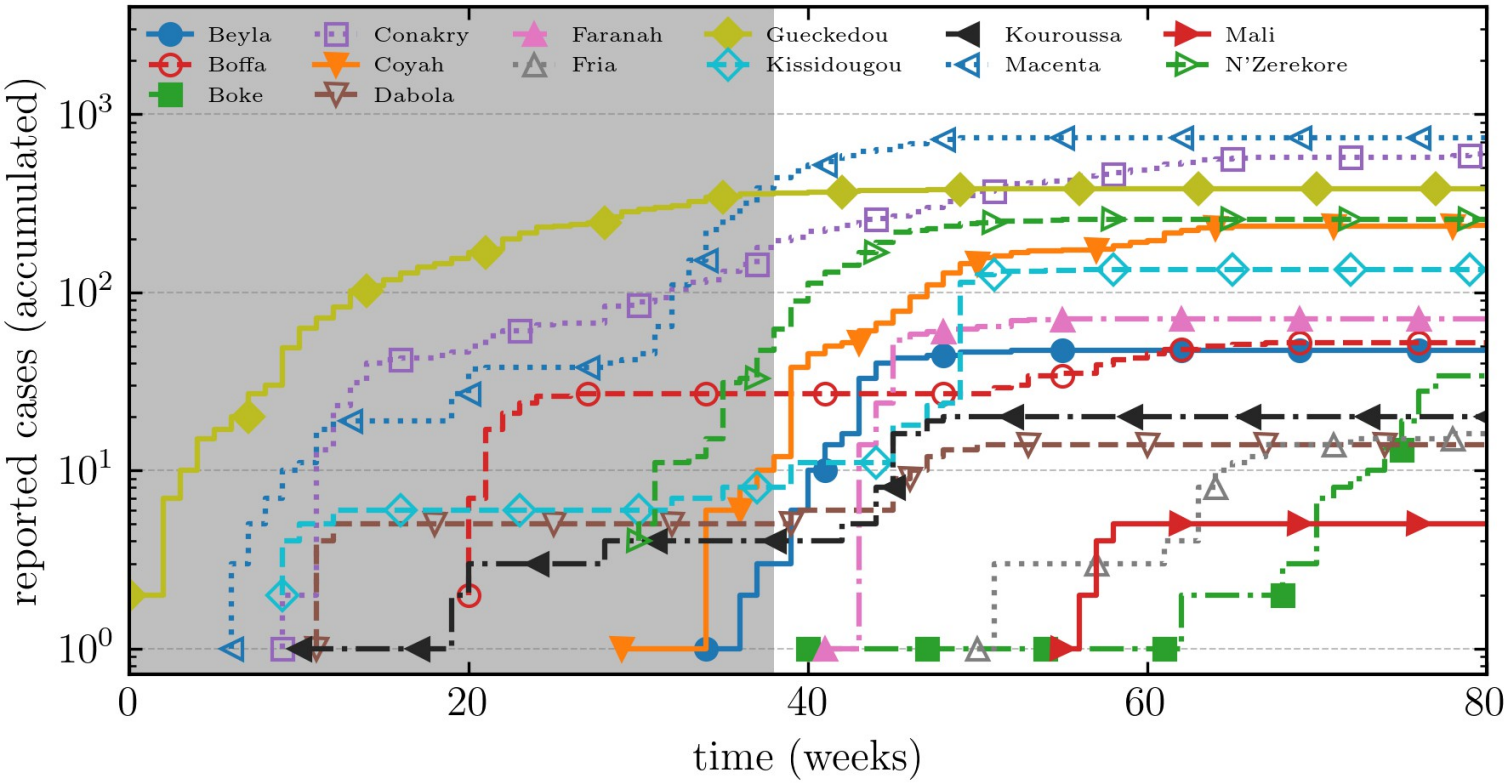
Ebola virus epidemic in Guinea. Shows the cumulative number of confirmed or probable cases at the district level. The time series begins on January 5, 2014, ends on May 8, 2016, and advances weekly increments. On August 8, 2014, several months after its beginning, the World Health Organization declared the outbreak in West Africa a Public Health Emergency of International Concern. On September 18, 2014, the United Nations established The United Nations Mission for Ebola Emergency Response (UNMEER). The grey shaded regions show the time before the creation of UNMEEER. Guinea was declared free of Ebola by the World Health Organization on June 1. We also show epidemic curves for 14 of 33 Guinean prefectures. The outbreak originated in the Guéckédou prefecture and quickly spread to Conakry, the capital city. Data from the World Health Organization.

Fig 1 shows the cumulative number of confirmed or probable cases at the district level in Guinea. The time-series begin on January 5, 2014, ends on May 8, 2016, and advances in weekly increments. The outbreak originated in Guinea, in the Guéckédou prefecture, and quickly spread to Liberia and Sierra Leone. On August 8, 2014, several months after its beginning, the World Health Organization declared the outbreak in West Africa a Public Health Emergency of International Concern. On September 18, 2014, the United Nations established The United Nations Mission for Ebola Emergency Response (UNMEER). The West African Ebola epidemic was declared over by the World Health Organization on June 9, 2016.

The local outbreaks in Fig 1 terminate at different times and the whole epidemic ends long before susceptible depletion. Before the United Nations convened to form UNMEER, many local outbreaks, including the outbreak in the Guéckédou prefecture where the epidemic originated, had already ceased. Naive epidemic theory predicts that onward transmission continues in the absence of interventions (e.g., contact tracing, isolation, or immunization) until herd immunity is reached. It is unclear if interventions or susceptible depletion can explain the abrupt termination of local outbreaks. Despite the large size of the outbreak compared to previous sporadic outbreaks of Ebola virus disease (EVD) in Sub-Saharan Africa, the total number of cases was also small compared to model projections [3]. Merler et al. modeled the spatial spread of Ebola virus disease in Liberia (2014) and concluded that Ebola treatment units (ETUs), safe burial procedures, and household protection kits explain the decrease in incidence [4, 5]. Here we explore an alternate hypothesis; that behavior changes explain the surprisingly small number of cases.

### 1.1 Human behavior and the 2014 West Africa Ebola outbreak

Ebola virus disease is transmitted by direct physical contact with infected bodily fluids [6]. According to the World Health Organization, avoiding direct contact with people who show Ebola symptoms reduces transmission. Asymptomatic infections are rare [7], suggesting that social distancing and isolation are effective in reducing the risk of human-to-human transmission. Burial rites are also a strong driver of Ebola transmission. Previous outbreaks in the Democratic Republic of Congo (DRC) and Uganda have shown that unsafe burial practices linger unless infection-control measures are adapted to local traditions [8]. Nonetheless, the 2014-2016 Ebola epidemic was massive compared to previously known outbreaks. An observational study found that of the cases exposed during funerals, 65% of those giving a response reported having touched the corpse. This proportion declined significantly after October 2014, suggesting that behavior changes had taken place [9]. Estimates of *R*_0_ during the 2014-2016 Ebola epidemic in West Africa are low, ranging between 1.2 and 2.2 [10]. Hence, a moderate reduction in transmissibility is sufficient to push *R*_0_ below the epidemic threshold [11].

It should be emphasized that the behavior changes mentioned above (i.e., avoiding direct contact with people who show Ebola symptoms, self-isolating, and not touching the corpse during a funeral or attending the ceremony) can occur autonomously. Findings by Drake et al. suggest behavior changes decreased the effective reproductive number in Liberia to almost one and that interventions further brought it down below the epidemic threshold [12]. Another study by Funk et al. found that healthcare-seeking behavior doubled throughout the outbreak in Lofa county, Liberia, but this was also linked to increased transmission inside treatment facilities [13]. The RAPID Ebola forecasting challenge compared the performance of eight independent modeling approaches on synthetic data and found that the top-performing models for short-term weekly incidence used reactive behavior changes [14].

## 2 Model

**Fig 2:**
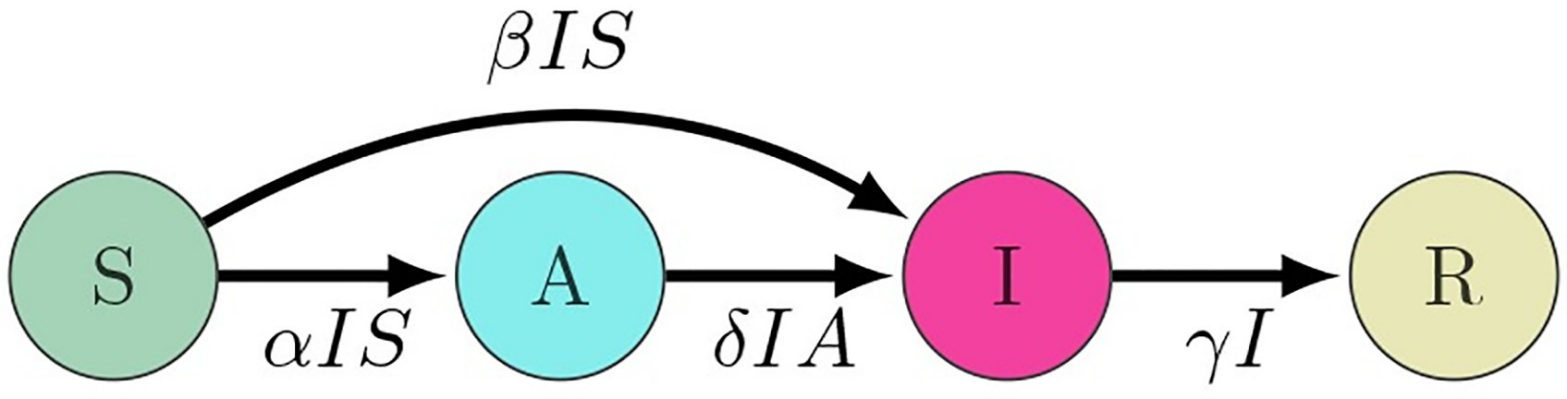
Diagram of the compartmental model. *β* is the contact rate, and *γ* is the rate of recovery. *α* is the rate of behavior change, and *δ* is the contact rate in the aware population.

Consider the following system of ordinary differential equations (ODE’s). Each equation represents a compartment of susceptible, aware, infectious, or removed agents [15].

$$\begin{array}{cc} \frac{dS}{dt} & =-\beta IS-\alpha IS, \end{array}$$ (1)

$$\begin{array}{cc} \frac{dA}{dt} & =\alpha IS-\delta AI, \end{array}$$ (2)

$$\begin{array}{cc} \frac{dI}{dt} & =\beta IS+\delta AI-\gamma I, \end{array}$$ (3)

where *β* is the contact rate, and *γ* is the rate of removal. *α* is the rate of behavior change, and *δ* is the contact rate for aware individuals. *A* is assumed to grow by a rate that is proportional to *I*, meaning that only symptomatic carriers can spread awareness. Fig 2 shows a diagram of the model.

Spatial heterogeneity was a distinguishing feature of the 2014-16 Ebola epidemic. However, the mean-field model above assumes homogeneous mixing. The West African Ebola epidemic has been the subject of many spatial modeling studies [4, 16–20]. Funk et al. showed that the impact of behavior change is more pronounced in the presence of spatial structure [21]. The tendency of local Ebola outbreaks to flare up and subside quickly suggests the epidemic could be locally self-organized. To explore this possibility, we will consider a spatial version of the mean-field model.

### 2.1 Spatial extension of the model

The spatial model is defined on a *L* × *L* lattice where one person occupies each site. A grid cell is either susceptible *S*, infectious *I*, aware *A* or removed *R*. Grid cells on the lattice are initialized in the susceptible state with a small number of infected sites to start the epidemic. Simulations run until infected have been removed. An asynchronous updating scheme is used with Δ*t* = 1.0 day equal to *L*^2^ updates. Do the following to perform an update.

a Select a random grid cell *s*_1_. If *s*_1_ is infected then proceed to (b). Else proceed to (c).
b
  1. Select another site *s*_2_. This site is chosen randomly on the lattice with probability *p*. Else with probability 1 − *p*, select *s*_2_ with a probability that decays exponentially with distance from *s*_1_. To be explicit, select a distance *d* with probability ∝exp(−*d*/λ) and choose a random point *s*_2_ at this distance.
  2. If *s*_2_ is susceptible, it is infected with probability *β*Δ*t*. Else if *s*_2_ is aware, it is infected with probability *δ*Δ*t*.
  3. Select another cell *s*_3_ as above. If *s*_3_ is susceptible, is becomes aware with probability *α*Δ*t*.
c *s*_1_ is removed with probability *γ*Δ*t*.

**Fig 3:**
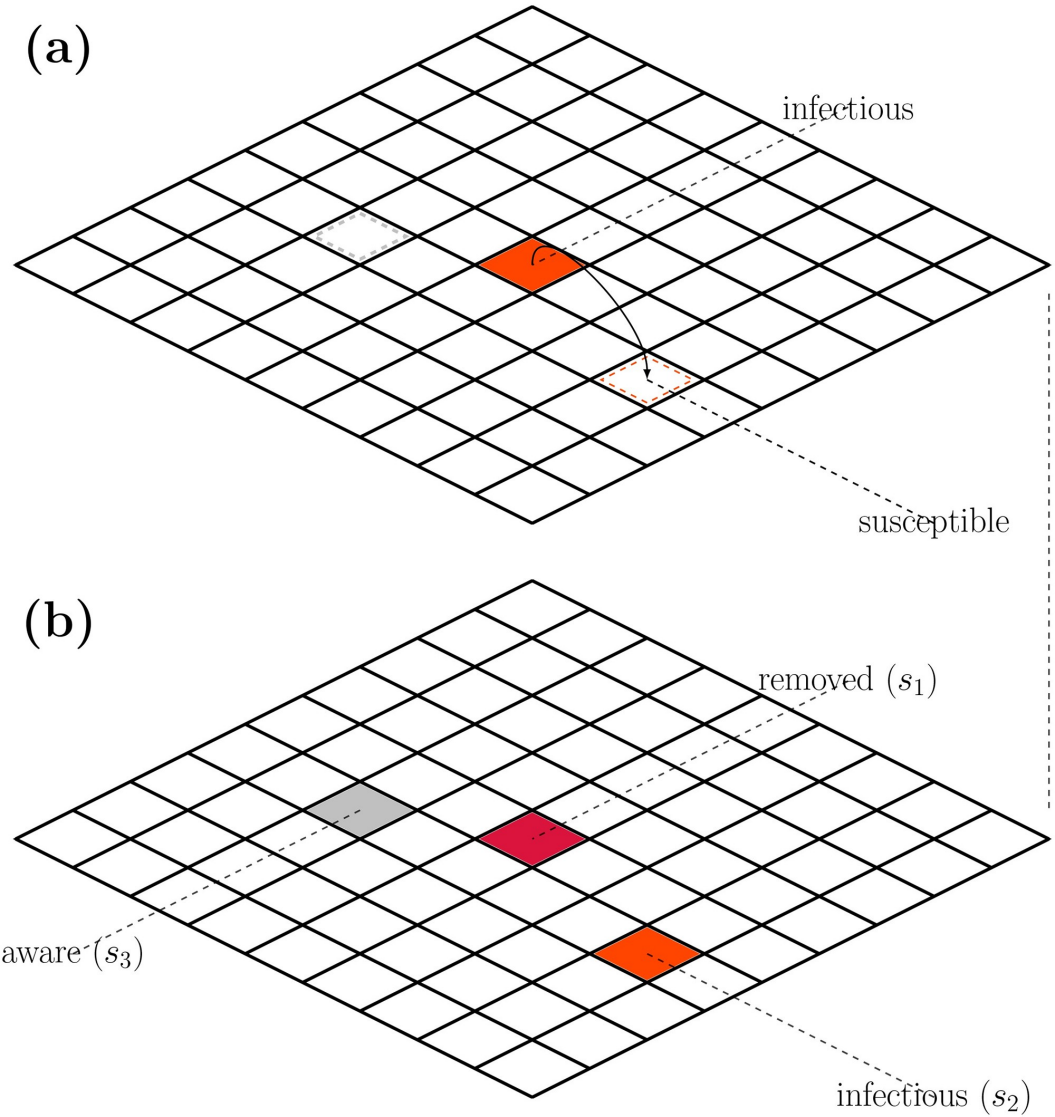
Diagram of the spatial model. Each site on the grid contains just one person. A small grid is with one infectious person in the center. (a) the infected person spreads disease and awareness to nearby squares. The transitions occur with probability *β* and *α*, respectively. The infected cell is removed with probability *γ*. (b) shows the updated grid.

Fig 3 shows a diagram of the spatial model. The extended model contains two spatial parameters, *p*, and λ in addition to the rate parameters *α*, *β*, *γ* and *δ*. There is a probability of selecting identical targets during an update i.e., *s*_2_ = *s*_3_. Since the reactions $S\overset{\beta }{\to }I$ and $S\overset{\alpha }{\to }\text{A}$ are mutually exclusive, it should be specified what happens in this event. Here we allow the reaction $S\overset{\beta }{\to }I$ to happen first. It is also possible to pick randomly between the two reactions, but the effect is completely negligible unless *α* and *β* are large. Global and local transmission allow for dispersal on different length scales. The effect of global transmission events is to seed spatially disassociated transmission clusters, which made up the epidemic in West Africa 2014-16 [22]. The previous known Ebola outbreaks have generally been localized, so this is a novelty. The effect of long-distance dispersal on epidemics has been studied by Shaw et al. [23, 24]. For *p* ∼ 1 or λ ∼ *L* we recover homogeneous mixing equivalent to the mean-field model.

## 3 Analysis

**Fig 4:**
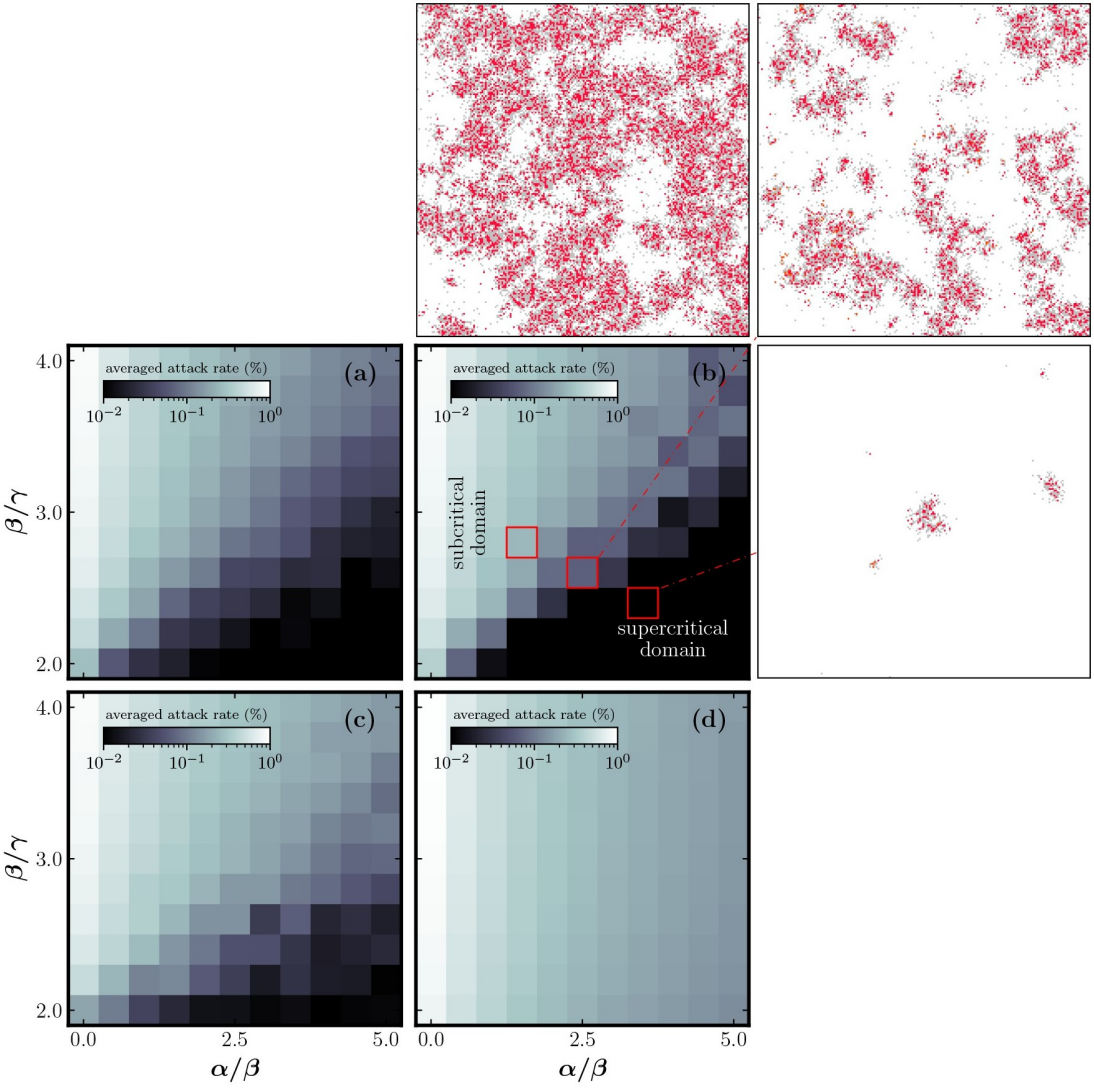
Phase diagram of the spatial model. The color shows the final attack rate (%). Outbreaks are controlled in the dark domain but spread unhindered in the light domain. (a) shows the spatial model on a 100 × 100 grid with *δ* = 0, *p* = 0.01 and λ = 2.0. (b) is simulated on a 200 × 200 grid, but otherwise identical to (a). Spatial configurations are shown for selected parameters, where all infected have been removed. The white grid cells are susceptible; grey cells are aware; red cells are removed. (c) Explores the effect of some susceptibility in the aware state, using *δ*/*γ* = 0.50 with other parameters as in (a). (d) shows homogeneous mixing with *p* = 1 and is otherwise identical to (a).

Fig 4 explores the effect of awareness transmission and idealized reproductive number. Successful spreading is a stochastic phenomenon, and many outbreaks die out early by chance [25]. The extinction probability is large when *I*(0) = 1 so we initiate our simulations with a cluster of *I*(0) = 16 infected sites placed in the center. To estimate the attack rate in Fig 4 we average over many simulations.

Outbreak size measured by the final attack rate increases with *β*/*γ* and decreases with *α*/*β*. The subcritical domain is carved out by the space of parameters where the disease is not contained. Outbreaks are rapidly enclosed by awareness in the supercritical domain. Fig 4(a) and 4(b) suggest that system size has a negligible effect. The attack rate is scale-invariant in the subcritical domain because the cluster spans the whole lattice. This scale-invariance is broken in the supercritical domain as the cluster grows to a finite size before it is enclosed by awareness. Here it is possible to reduce the attack rate by scaling the system.

Panel (c) shows the effect of nonzero *δ*, which is moderate provided that *δ* is not increased above the critical value where transmission is sustainable in a fully aware population.

Panel (d) reveals the absence of a supercritical state in a well-mixed system. It is no longer possible for awareness to spread around and enclose a cluster of infected cells; therefore, it does not affect the epidemic threshold [21]. The effect of susceptible depletion is also decreased as infected grid cells face much less intraspecific competition.

### 3.1 Heterogeneity measures

**Fig 5:**
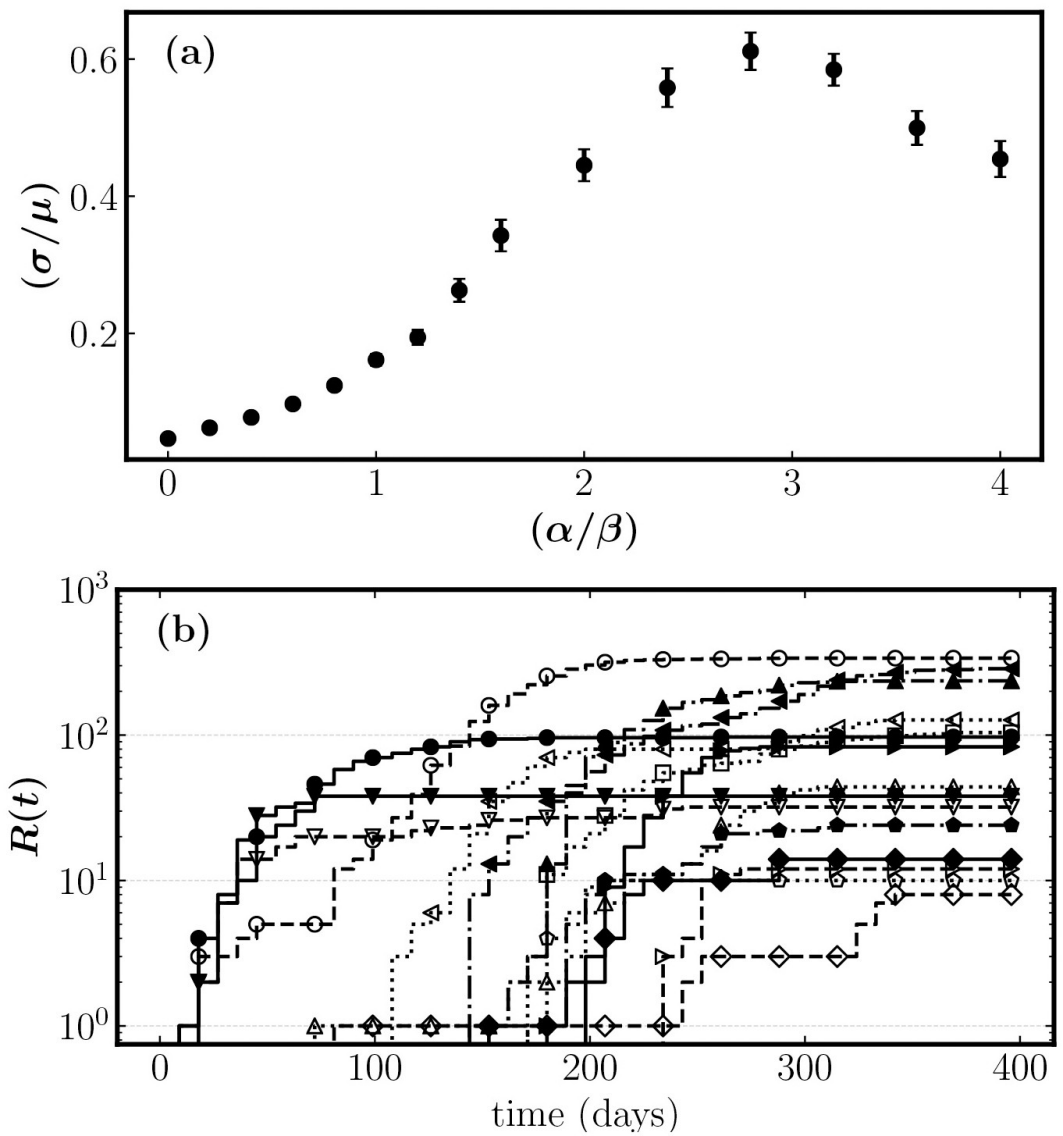
(a) shows the coefficient of variation *c*_*v*_ between the subgrids for increasing *α*. Model parameters: *β* = 0.25, *γ* = 0.10, *δ* = 0, and *p* = 0.01. The scale parameter in the exponential distribution was 2.0. Grid size was 200 × 200. (b) shows removed cases *R*(*t*) for each subgrid in a simulation around criticality *α* = 0.70 with parameters as above.

The boundary between the sub and supercritical domain is particularly interesting. The epidemic can often seed multiple spatially dissociated transmission clusters before containment, resulting in a high degree of spatial heterogeneity. The size variation among the clusters can range from a few infected to clusters spanning the whole system. We explore this by dividing a 200 × 200 lattice into subgrids of 50 × 50 grid cells. Variations between the subgrids can be used to measure spatial heterogeneity. Fig 5(a) shows the standard deviation to mean ratio *c*_*v*_ of the 16 subgrids. The dispersion is low before criticality; the cluster will span the whole lattice producing only minor variations between the subgrids. Spatial heterogeneity is maximized around criticality where some regions have massive outbreaks and others none at all. The region-to-region variability drops in the supercritical state where outbreaks are rapidly contained.

Fig 5(b) shows the number of cases over time in each subgrid with critical awareness spreading. Features are comparable to Fig 1 distinguished by subexponential growth and outbreaks of varying sizes that saturate rapidly before susceptible depletion.

## 4 Discussion

The model can explain the spatial patterns observed during the 2014-16 epidemic in Guinea. However, many assumptions have been made throughout the paper. The rate of behavior change *α* and its effect *δ* are not derived from data. More data is needed to understand the dynamic interplay between infection and human behavior. Evidence from the ongoing SARS-CoV-2 pandemic suggests that behavior changes have a significant effect on disease transmission. However, the risk of Ebola infection is likely to induce more drastic behavior changes than COVID-19, given the vast discrepancy in the case fatality ratio. Data concerning Ebola-related behavior change is limited. It is known that participation in risky funerals declined over time in Guinea, Liberia, and Sierra Leone. The International Ebola Response Team further found that this decline was positively correlated with the within-district transmission intensity, supporting the proposition that local prevalence drives behavior changes [9]. Awareness is more likely to be present in communities with either ongoing transmission or past exposure. However, awareness is expected to fade over time as the perceived risk of infection decreases. Our model does not contain a fading term, but adding one is not difficult. Glaubitz et al. found that this could cause oscillatory behavior in a homogeneous mixing model [26]. The presence of spatial structure should have a dampening effect on such oscillations because different regions quickly come out of phase. If a localized outbreak ends and the remaining *A* states are returned to *S* states, there are no infected to spread the disease. It is, therefore, necessary to introduce the disease again, either through a global transmission event or by encroachment from another growing transmission cluster.

Precise estimates of the susceptibility of aware people *δ* are unnecessary; it suffices to show that the disease cannot survive in a fully aware population. Estimating *δ* is difficult because humans can exhibit a range of behaviors that produces a spectrum of *A* states with corresponding contact rates. However, it is possible to identify specific behaviors with a significant effect. Lagrand’s model breaks the reproduction number down into components that can be ascribed to various settings, including community *R*_*c*_, hospitals *R*_*h*_, and funerals *R*_*F*_ [5]. The *R*_*F*_ component is driven by behavior and gives a lower bound on the effect of awareness. The weight carried by each of these terms can vary significantly between Ebola outbreaks, suggesting that *δ* is very outbreak-specific.

The spatial model is simulated on a grid with one idealized person in each grid cell. While this is not realistic, the effect of population density is unlikely to be profound. Hu et al. has estimated how contact rates scales with population density [27, 28]. However, symptomatic Ebola patients are likely to be bedridden during infection. Those most likely of exposure are caring family members, health care personal, or those attending funerals. Accordingly, the rates could be largely independent of population density.

The SARS-CoV-2 pandemic has shown us that variations in behavior explain many differences between countries. Many factors influence how people respond, including the risk of infection, age, and compliance with public health guidelines. Nonetheless, the latter will carry more weight in countries with high trust in government. Ebola outbreaks have so far happened in countries where trust in government is low. Here the behavior response is more likely to be a local effect because people respond to community transmission more than recommendations from public health officials. We have shown how such a local behavior response can explain the abrupt termination of local outbreaks, as was observed during the 2014-2016 Ebola epidemic.
